# New investigation of encoding secondary metabolites gene by genome mining of a marine bacterium, *Pseudoalteromonas viridis* BBR56

**DOI:** 10.1186/s12864-024-10266-6

**Published:** 2024-04-13

**Authors:** Desy Putri Handayani, Alim Isnansetyo, Indah Istiqomah

**Affiliations:** Department of Fisheries, Faculty of Agriculture, Universitas Gadjah Mada, Yogyakarta, Indonesia

**Keywords:** AntiSMASH, Bagel4, Bioinformatic, 16S rDNA, Chromosome

## Abstract

*Pseudoalteromonas viridis* strain BBR56 was isolated from seawater at Dutungan Island, South Sulawesi, Indonesia. Bacterial DNA was isolated using Promega Genomic DNA TM050. DNA purity and quantity were assessed using NanoDrop spectrophotometers and Qubit fluorometers. The DNA library and sequencing were prepared using Oxford Nanopore Technology GridION MinKNOW 20.06.9 with long read, direct, and comprehensive analysis. High accuracy base calling was assessed with Guppy version 4.0.11. Filtlong and NanoPlot were used for filtering and visualizing the FASTQ data. Flye (2.8.1) was used for de novo assembly analysis. Variant calls and consensus sequences were created using Medaka. The annotation of the genome was elaborated by DFAST. The assembled genome and annotation were tested using Busco and CheckM. Herein, we found that the highest similarity of the BBR56 isolate was 98.37% with the 16 S rRNA gene sequence of *P. viridis* G-1387. The genome size was 5.5 Mb and included chromosome 1 (4.2 Mbp) and chromosome 2 (1.3 Mbp), which encoded 61 pseudogenes, 4 noncoding RNAs, 113 tRNAs, 31 rRNAs, 4,505 coding DNA sequences, 4 clustered regularly interspaced short palindromic repeats, 4,444 coding genes, and a GC content of 49.5%. The sequence of the whole genome of *P. viridis* BBR56 was uploaded to GenBank under the accession numbers CP072425–CP072426, biosample number SAMN18435505, and bioproject number PRJNA716373. The sequence read archive (SRR14179986) was successfully obtained from NCBI for BBR56 raw sequencing reads. Digital DNA–DNA hybridization results showed that the genome of BBR56 had the potential to be a new species because no other bacterial genomes were similar to the sample. Biosynthetic gene clusters (BGCs) were assessed using BAGEL4 and the antiSMASH bacterial version. The genome harbored diverse BGCs, including genes that encoded polyketide synthase, nonribosomal peptide synthase, RiPP-like, NRP-metallophore, hydrogen cyanide, betalactone, thioamide-NRP, Lant class I, sactipeptide, and prodigiosin. Thus, BBR56 has considerable potential for further exploration regarding the use of its secondary metabolite products in the human and fisheries sectors.

## Introduction

Research on terrestrial natural product compounds has been extensively performed and has succeeded in exploring, isolating, and purifying compounds that are important for human health. Studies on potential sources other than terrestrial are of interest to researchers, especially those from the marine ecosystem [1]. Marine ecosystems hold considerable potential for natural resources that are beneficial for many purposes. Seawater is a large reservoir for microorganisms, including bacteria, from which secondary metabolite compounds can be isolated. Natural products from the marine environment have different character structures compared with compounds isolated from the terrestrial environment [2]. Secondary metabolite compounds are produced by bacteria to counter environmental changes in biological, chemical, and physical terms. The bioactivity of secondary metabolite compounds, including antibacterial, antifungal, antiparasitic, anticancer, antioxidant, antifouling, and algicidal compounds, has been widely studied [3–5]. The genus *Pseudoalteromonas* contains marine bacteria that can produce secondary metabolite compounds [6].

*Pseudoalteromonas* belongs to the order Alteromonadales in the Gammaproteobacteria class. These bacteria can produce various natural compounds that can be used for antibiotics, antifungal, antibiofouling, and anticancer purposes [3, 7–9]. *Pseudoalteromonas* are Gram negative, aerobic, and motile bacteria and do not produce spores but do require sea water to live and grow optimally. Studies related to *Pseudoaltermonas* have been performed since the 20th century, and in 1995, Gauthier separated the genus *Pseudoalteromonas* from *Alteromonas* [10]. stated that 49 species of *Pseudoalteromonas* have been recorded to date, one of which is *Pseudoalteromonas viridis*. Studies related to the potential production and use of secondary metabolites from bacteria can be conducted using two methods. The first method uses in vitro bioactivity screening. Bacteria are grown on media, isolated, and bioassayed, and any relevant compounds identified. This method takes a long time and only detects 1–2 compounds in one process. Another more effective and efficient method to investigate bioactive compounds is genome mining. Genome mining can more quickly investigate the potential of genes encoding natural products so that bacterial growth can be manipulated, or stressors can be applied to obtain targeted compounds [11]. revealed that whole bacterial genomes can be analyzed using various whole genome sequencing (WGS) methods, including Oxford Nanopore Technology, Illumina, Roche 454, and PacBio. Many valuable bioactive compounds have since been discovered through bacterial genome mining.

WGS studies on *Pseudoalteromonas* species have been conducted on *P. tunicata, P. piscicida, P. agarivorans, P. atlantica*, and *P. xiamenensis* [6, 12–16]. However, few studies have been performed on *P. viridis* and its potential to produce natural product compounds. Herein, this study revealed that *P. viridis* BBR56 has antibacterial activity against *Vibrio* sp. This study also investigated WGS for *P. viridis* BBR56 genome mining purposes regarding genes encoding bioactive compounds through biosynthetic gene cluster (BGC) analysis. In addition, the genome of *P. viridis* BBR56 was compared with those of other *Pseudoalteromonas* species to determine differences in the characteristics of the encoded genes.

## Materials and methods

### Culture media and morphological identification for bacteria

*P. viridis* BBR56 was isolated from the seawater of Dutungan South Sulawesi, Indonesia by the pour plate microdilution method. Zobell 2216E agar media was used to grow bacterial cells and contained 15 gL^− 1^ bacteriological agar, 1 gL^− 1^ yeast extract, and 5 gL^− 1^ peptone in water (20 ppt; pH 7.5). Zobell 2216E broth medium was prepared by mixing the same ingredients without adding agar. Bacterial medium sterilization was performed in an autoclave (121 °C for 15 min, 15 psi). The bacterial sample was purified using Zobell 2216E medium to obtain single colonies. Morphological and simple biochemical tests were performed based on the methodology of [17]. Observation of BBR56 cell morphology was assessed using KOH 3% and Gram-staining analysis. A simple test was elaborated to analyze catalase, oxidase, and motility activity. For further study, bacteria were inoculated on broth medium containing glycerol and stored at − 80 °C [18].

### Antibacterial activity evaluation

*P. viridis* BBR56 was inoculated and incubated in Zobell 2216E broth medium for 96 h at room temperature in an orbital shaker (Daihan Scientific SHO-2D, South Korea) with shaking at 120 rpm. The bacterial culture was centrifuged at 3,500 *g* for 50 min to separate the supernatants and pellets. Whatman no 1 filter paper was used for filtering the supernatant to obtain cell-free supernatant (CFS). Next, ethyl acetate was used to extract the CFS, and the pellets were extracted with ethanol. Samples were then sonicated using an ultrasonicator (US-300T, Japan) for 90 min [17]. CFS and pellet extracts of *P. viridis* BBR56 were evaluated for inhibiting the growth of pathogenic *V. harveyi* BT1H (accession number LN610442). In this study, we used 5% ethanol as a negative control and enrofloxacin as a positive control. The bacterial growth-inhibiting activity was evaluated by the paper disk (8-mm diameter) diffusion method on double-layered agar [19]. The bacterial activity was checked by measuring the diameter of the inhibition zone.

### Molecular identification of *P. Viridis* BBR56

DNA was isolated from *P. viridis* BBR56 using a DNA extraction kit (Promega Genomic DNA, Wizard, USA). The 16S rRNA gene was amplified with universal primers 27F 5’-AGAGTTTGATCMTGGCTCAG-3’ and 1492R 5’-CGGTTACCTTGTTACGACCTT-3’ was performed using a thermal cycler machine (Bio-Rad T100) [20–22]. The thermal cycling conditions were 95 °C for 3 min, 94 °C for 30 s, 55 °C for 30 s, 72 °C for 90 s (32 cycles), and a final extension for 5 min at 72 °C. The PCR product was then sequenced by PT Genetika Science Indonesia for 16 S rRNA gene. The sequences of the samples were then analyzed and aligned by Bioedit then checked for homology search of 16 S rRNA gene by BLAST [23]. The highest percentage of similarity was chosen as the species similar to the sample sequence. More than 32 bacterial 16 S rRNA gene sequences were chosen to construct a neighbor-joining phylogeny tree using MEGA7 [24–26].

### Sequencing and assembly of the bacterial genome

Preparation of the DNA library was conducted by Oxford Nanopore Technology (PT Genetika Science, Indonesia), GridION MinKNOW 20.06.9. Guppy version 4.0.11 was used for base calling, according to [27]. Filtlong was used to filter the FASTQ file after base calling (https://github.com/rrwick/Filtlong), and the DNA quality was visualized via NanoPlot [28] (de Coster et al. 2018). Then, de novo assembly was performed using Flye 2.8.1 [29]. Variant calls and consensus sequences were created using Medaka. The annotation of the genome was elaborated by DFAST [30, 31] mentioned that the sequence of the assembled genome was analyzed by Busco.

### Annotation and comparative genome analysis

The *P. viridis* BBR56 genome was annotated by using CheckM as in [32]. Trapid software analyzed functional genes for several purposes, including the Kyoto Encyclopedia of Genes and Genomes Orthologous dataset and Gene Ontology (GO). Orthologous Groups Cluster of Proteins (COG) was analyzed using EggNOG [33]. Genes encoding secondary metabolites were investigated using antiSMASH 6.0 (https://antismash.secondarymetabolites.org) [34]. We also BAGEL4 (http://bagel4.molgenrug.nl/) [35]. To analyze the potential for bacteriocin production from genes encoded in this bacterial genome, genome comparison was performed using the genome data of BBR56, *P. maricaloris, P. rubra*, *P. piscicida*, and *P. flavipulchra*, which were taken from the NCBI. OrthoVenn2 was used for determining the comparison and annotation of the bacterial genome (https://orthovenn2.bioinfotoolkits.net/) as described by [36].

### Digital DNA–DNA hybridization

The genome sequence data of *P. viridis* BBR56 were uploaded to the Type Strain Genome Server (TYGS) available under https://tygs.dsmz.de, a free bioinformatics platform [37]. TYGS sister data provides information regarding synonymy, nomenclature, and associated taxonomic literature (available at https://lpsn.dsmz.de). A minimum evolution phylogenetic tree with intergenomic distances and SPR postprocessing was built using FASTME 2.1.6.1 [38]. The branch of the phylogenetic tree was analyzed from more than 100 pseudobootstrap replicates, and PhyD3 was used to visualize the tree [39].

## Results

### Antibacterial activity

**Fig. 1:**
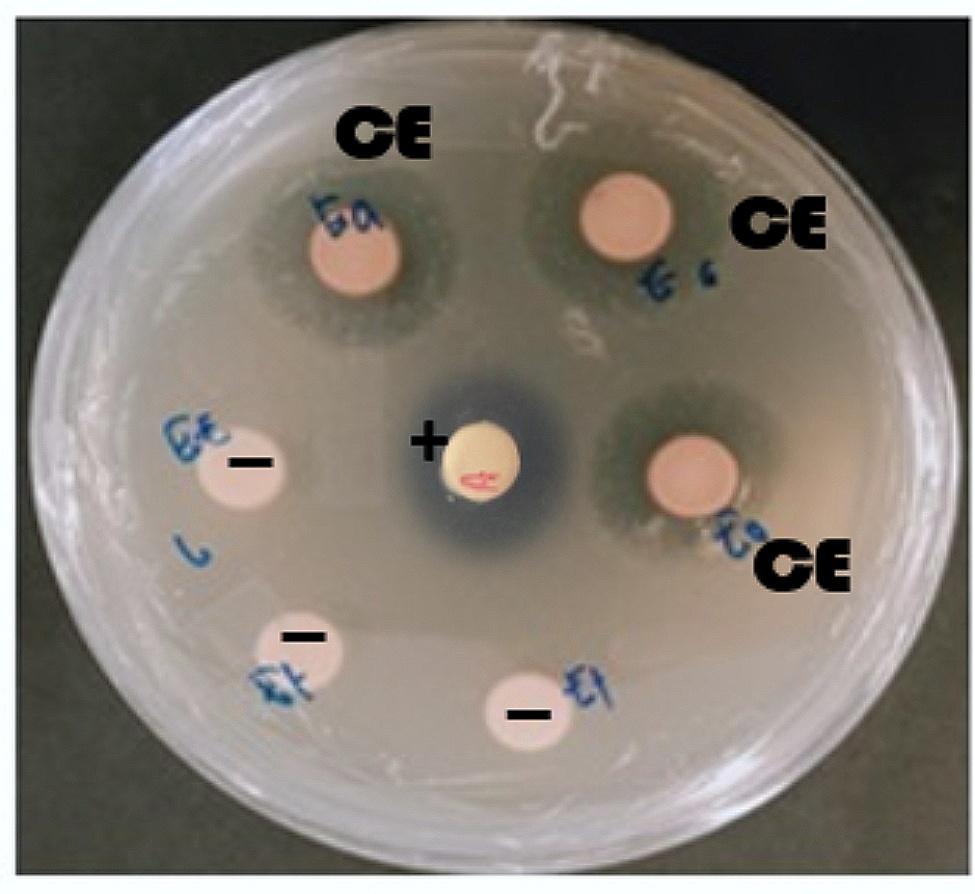
Antibacterial activity of supernatant extracts from *P. viridis* BBR56 at 2,500 µg/disc against pathogenic *V. harveyi* BT1H

No antibacterial activity was present in the pellet extract. The supernatant extracts from *P. viridis* BBR56 at 2,500 µg/disc had an antibiotic mechanism against *V. harveyi* BT1H with diameter of inhibition zone of 15.67 ± 0.58 mm (Fig. 1). This result revealed that *P. viridis* BBR56 could produce a potent antibacterial substance.

### Morphological and molecular identification

**Fig. 2:**
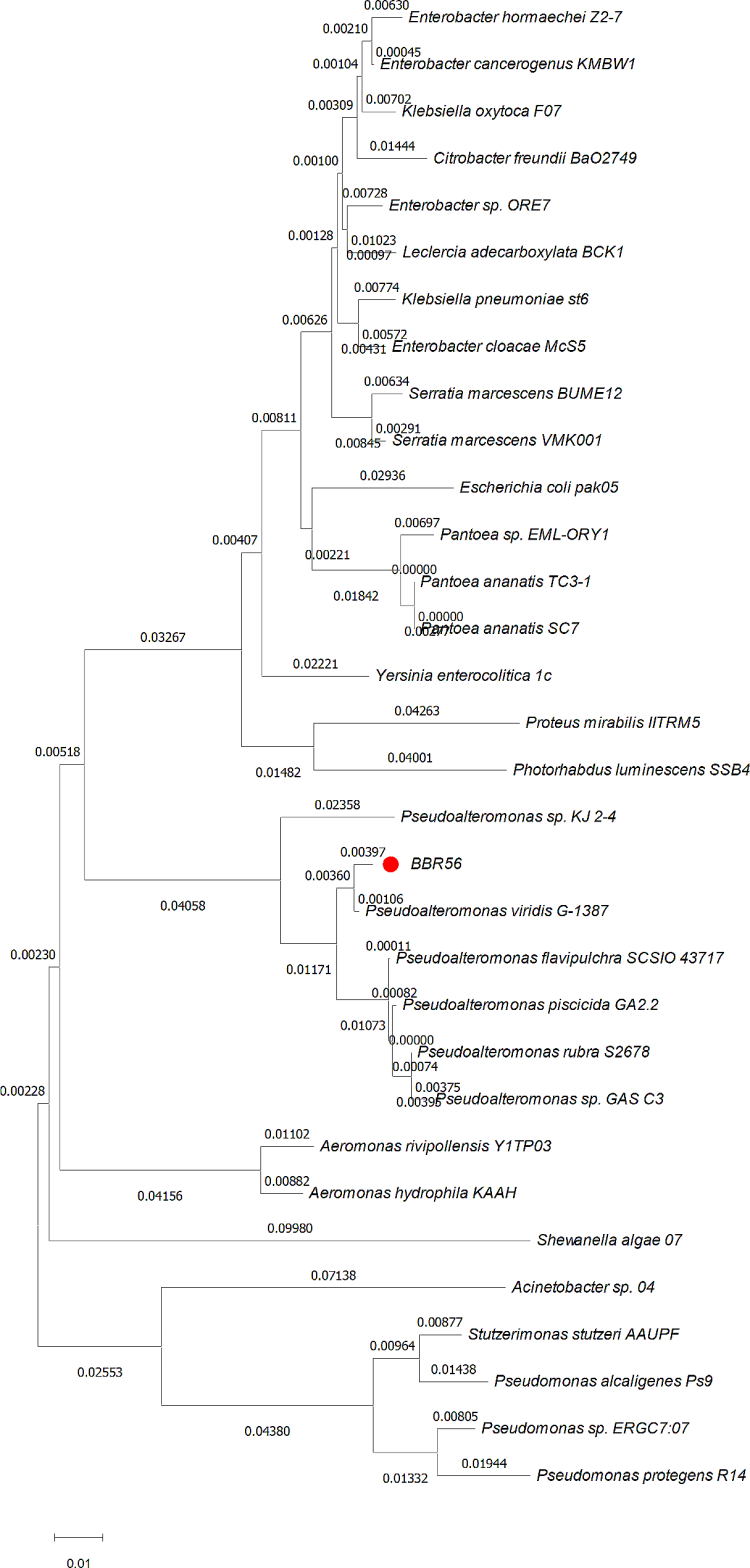
Phylogenetic relationships built upon the 16 S rRNA gene sequences of BBR56 by neighbor-joining analysis

Colonies of BBR56 had a bright red color, and *P. viridis* BBR56 was classified as Gram negative, motile, and oxidase and catalase-positive bacterium. Molecular identification showed that *P. viridis* BBR56 was closely related to *P. viridis* G1387, *P. rubra* 1943, and *Pseudoalteromonas* sp. was 98.37%, 98.09%, and 92.34%, respectively. The phylogenetic tree analysis showed that BBR56 was most closely related to *P. viridis* G1387 (Fig. 2).

### Genome features

**Fig. 3:**
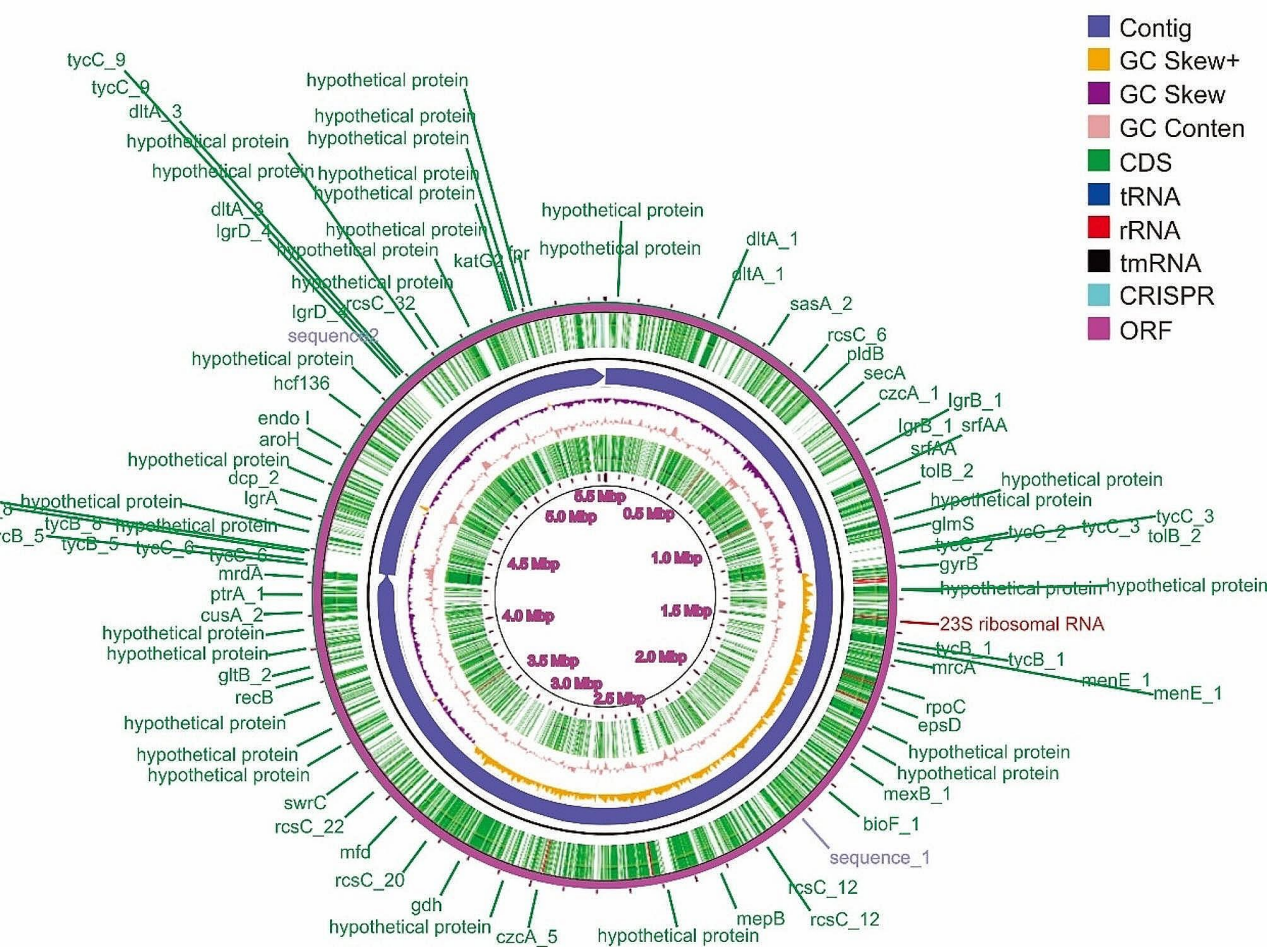
The circular *P. viridis* BBR56 genome consisting of two chromosomes was constructed using CG View Server Beta (http://cgview/ca)

The BBR56 strain was isolated from seawater, and the genome size was 5.5 Mb and included chromosome 1 (4.2 Mbp) and chromosome 2 (1.3 Mbp); these contained 61 pseudogenes, 4 noncoding RNAs, 113 tRNAs, 31 rRNAs, 4,505 coding DNA sequences, 4 clustered regularly interspaced short palindromic repeats, and 4,444 coding genes and had a 49.5% GC content. COG data showed that 21.70% of proteins had unknown function, 2.34% were used for defense mechanism, and 2.39% were used for transport gene, secondary metabolites, and catabolism. The circular presentation of the of *P. viridis* BBR56 genome is presented in Fig. 3. The whole genome, biosample, and bioproject of BBR56 were uploaded to GenBank under Accession Numbers CP072425–CP072426, SAMN18435505, and PRJNA716373, respectively. The sequence read archive (SRR14179986) was successfully obtained from NCBI for BBR56 raw sequencing reads.

### Digital DNA–DNA hybridization

**Fig. 4:**
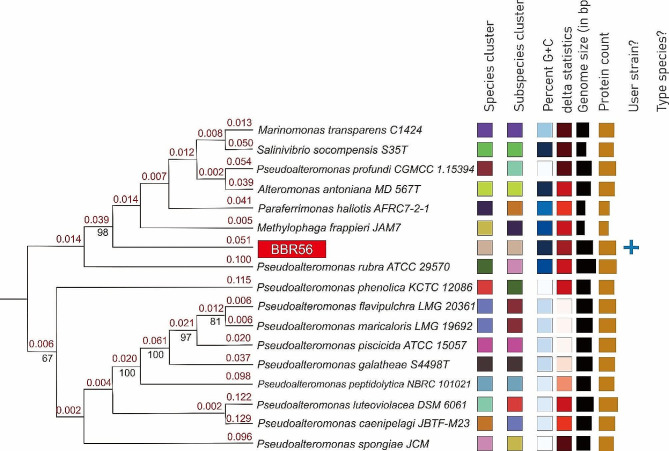
Genome phylogenetic tree of BBR56 compared with other bacterial genomes using FastME 2.1.6.1 from GBDP distances calculated from genome sequences. Branch value was assessed from 100 replications, with an average branch support of 48.9, using GBDP pseudobootstrap support values > 60%

The digital DNA–DNA hybridization analysis showed that the BBR56 genome could be a new species because no other bacterial genome was found to be similar to the sample. The phylogenetic tree of the genome used for dDDH analysis is shown in Fig. 4. The BBR56 genome pairwise comparison with other bacterial genomes is shown in Table 1, which contains the pairwise dDDH values of the BBR56 genome and the selected strain genomes. The confidence interval was shown together with the dDDH values for the three different Genome BLAST Distance Phylogeny approach (GBDP) formulas: d_0_ was calculated by dividing the length of all HSPs (high scoring segment pairs) by the total genome length, d_4_ was calculated by dividing the amount of all identities by the overall HSP length, and formula d_6_ was calculated by dividing all identities by the total genome length.

**Table 1:** Pairwise comparisons of BBR56 genomes with other bacterial genomes by digital DNA–DNA hybridization analysis

Query	Subject	d_0_	C.I. d_0_	d_4_	C.I. d_4_	d_6_	C.I. d_6_	Different G + C content
BBR56	*Marinomonas* *transparens* C1424	12.8	[10.1– 16.0]	49.0	[46.4– 51.6]	13.2	[10.8– 16.0]	5.43
BBR56	*Methylophaga frappieri* JAM7	12.9	[10.2–16.1]	43.5	[40.9–46.0]	13.3	[10.9–16.1]	1.34
BBR56	*Paraferrimonas haliotis* AFRC7-2-1	12.9	[10.2–16.2]	42.4	[39.9–45.0]	13.3	[11.0–16.1]	2.1
BBR56	*Alteromonas antonina* MD_567T	12.9	[10.2–16.2]	36.0	[33.5–38.5]	13.3	[11.0–16.1]	0.4
BBR56	*Salinivibrio sonomensis* S35T	12.9	[10.2–16.2]	32.8	[30.4–35.3]	13.3	[11.0–16.1]	0.25
BBR56	*P. spongiae* JCM 12,884	13.2	[10.5–16.5]	24.8	[22.4–27.2]	13.6	[11.2–16.3]	8.45
BBR56	*P. profundi* CGMCC 115,394	13.8	[11.0–17.1]	24.2	[21.9–26.7]	14.1	[11.7–16.9]	7.79
BBR56	*P. caenipelagi* JBTF-M23	14.2	[11.4–17.6]	20.5	[18.3–22.9]	14.4	[12.0–17.3]	7.28
BBR56	*P. peptidolytica* NBRC 101,021	14.3	[11.5–17.7]	20.4	[18.2–22.8]	14.5	[12.1–17.4]	6.82
BBR56	*P. phenolica* KCTC 12,086	14.4	[11.5–17.8]	21.6	[19.3–24.0]	14.6	[12.2–17.5]	8.68
BBR56	*P. piscicida* ATCC 15,057	14.6	[11.8–18.0]	19.7	[17.5–22.1]	148	[12.3–17.6]	6.04
BBR56	*P. flavipulchra* LMG 20,361	14.8	[11.9–18.2]	20.3	[18.1–22.7]	14.9	[12.4–17.8]	6.02
BBR56	*P. maricaloris* LMG 19,692	14.8	[11.9–18.2]	20.2	[18.0–22.6]	14.9	[12.4–17.8]	6.15
BBR56	*P. galathea* S4498T	14.9	[12.1–18.4]	21.0	[18.7–23.4]	15.1	[12.6–17.9]	6.27
BBR56	*P. luteoviolacea* DSM 6061	15.2	[12.3–18.7]	19.5	[17.3–21.9]	15.3	[12.8–18.2]	7.49
BBR56	*P. rubra* ATCC 29,570	56.9	[53.4–60.4]	25.9	[22.4–27.2]	46.8	[43.8–49.8]	1.47

### Genome comparison of *P. Viridis* BBR56

**Fig. 5:**
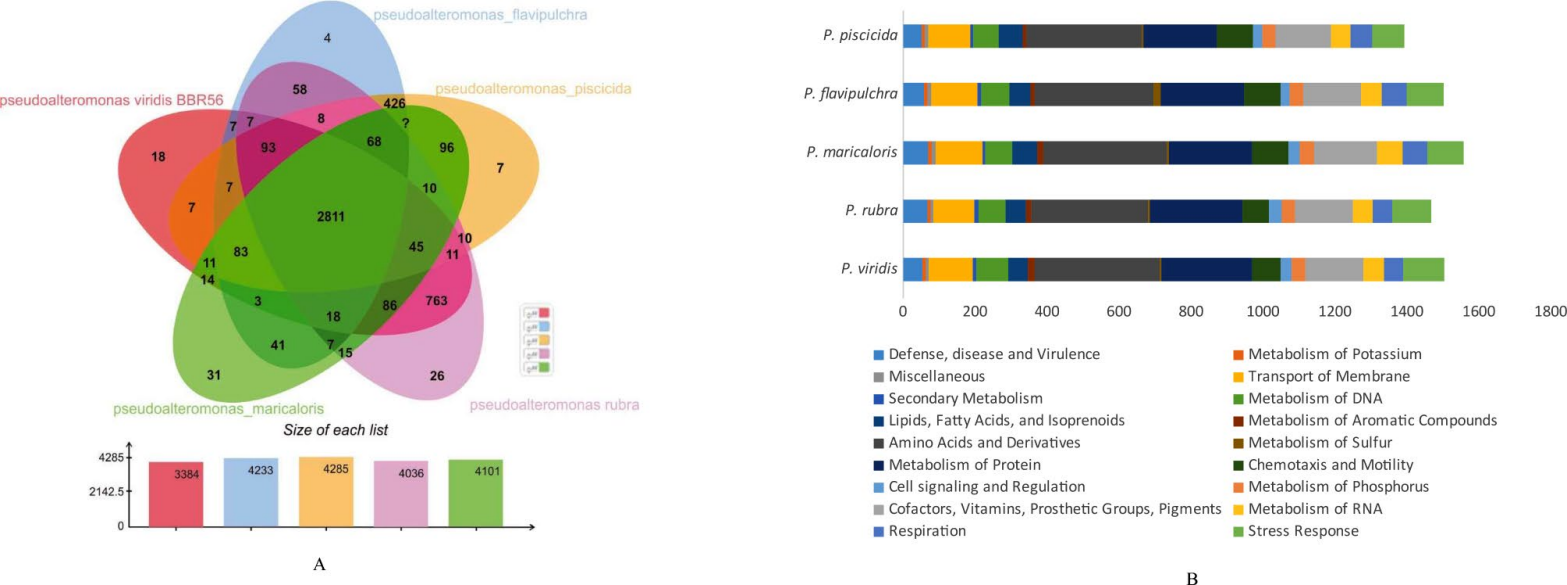
Comparison of the genome of *P. viridis* BBR56 with genomes of other *Pseudoalteromonas* species. (A) Venn diagram of characteristic gene clusters between *P. viridis* BBR56 (pink), *P. maricaloris* (green), *P. rubra* (purple), *P. flavipulchra* (blue), and *P. piscicida* (orange). (B) Analysis subsystem of biological processes from five genomes, shown by a bar graph of 18 different categories

The genome of *P. viridis* BBR56 was found to be most similar to that of *P. maricaloris*, *P. flavipulchra*, *P. piscicida*, and *P. rubra* based on the analysis of the genome phylogeny tree using TYGS data. The genome size of *P. viridis* BBR56 was 5.5 Mbp, whereas that of *P. maricaloris*, *P. rubra*, *P. flavipulchra*, and *P. piscicida* was 5.5, 6.1, 5.4, and 4.2 Mbp, respectively. Genomic comparison via OrthoVenn2 analysis of *P. viridis* BBR56 and the genome of other *Pseudoalteromona*s species showed that the five genomes formed 2,611 orthologous clusters, 2,772 single-copy gene clusters, and 5,383 protein clusters. A total of 2,822 protein clusters were shared by all genomes while *P. viridis* BBR56 shared 7, 763, 14, and 11 clusters with *P. flavipulchra*, *P. rubra*, *P. maricaloris*, and *P. piscicida*, respectively. A total of 86 protein clusters were identified for all single genomes, with 18, 4, 7, 26, and 31 clusters present in *P. viridis* BBR56, *P. flavipulchra, P. piscicida, P. rubrawere*, and *P. maricaloris*, respectively (Fig. 5a). Analysis of the subsystem with RASTk revealed that a large portion of the biological processes encoded by those bacterial genomes were required for amino acids and derivatives, whereas the secondary metabolite genes only represented 0.5% of the genomes (Fig. 5b).

### Antibiotic BGCs

Secondary metabolite coding genes were investigated using antiSMASH for bacteria and BAGEL4, which detected 17 regions on the two chromosomes (10 regions on chromosome 1 and 7 regions on chromosome 2) contained in the *P. viridis* BBR56 genome. From 15 regions, genes were identified for polyketide synthase, nonribosomal peptide synthase, RiPP-like, NRP-metallophore, hydrogen cyanide, betalactone, thioamide-NRP, Lant class I, sactipeptide, and prodigiosin. The BAGEL4 analysis showed that two types of bacteriocin were detected in the genome: antipeptide class I and sactipeptide.

### RiPP-like gene

**Fig. 6:**
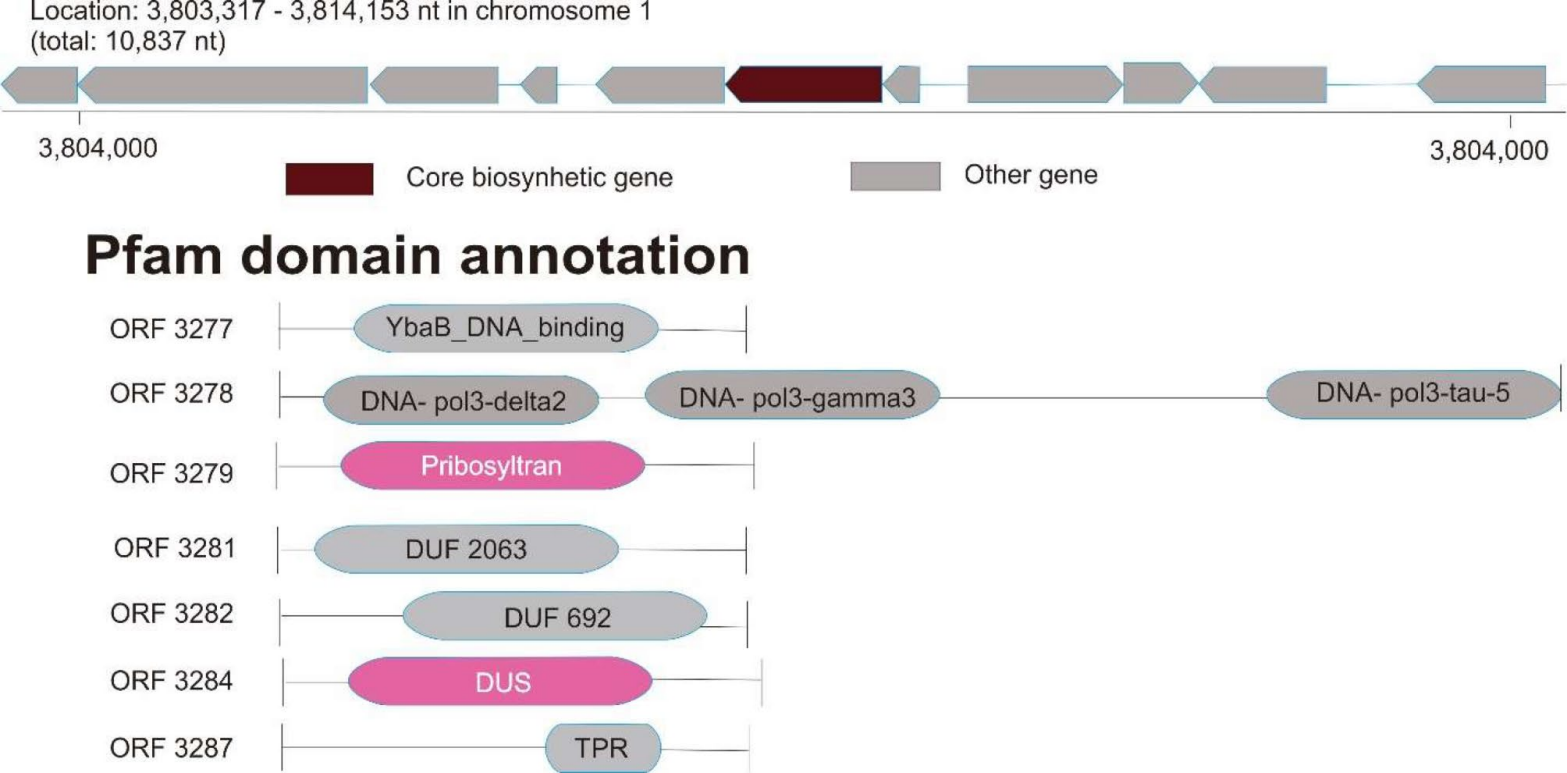
Visualization of the RiPP-like biosynthetic gene cluster of *P. viridis* BBR56 using antiSMASH, bacterial version (https://antismash.secondarymetabolites.org)

We identified a RiPP-like gene in the region 3.803.317–3.814.153 of chromosome 1 (10.837 nt). The biosynthetic rule-based cluster of RiPP-like was DUF692 (Fig. 6).

### Nonribosomal peptide synthase (NRPS)

NRPS genes were identified in several regions in chromosomes 1 and 2 of the *P. viridis* BBR56 genome. NRPS in region 1.4 and contained condensation with AMP-binding acting as the core biosynthetic gene; PP-binding and PF00561 as additional genes; SMCOG1197: autoinducer-binding transcriptional regulator as a regulatory gene, and the SMCOG1049: AcrB/AcrD/AcrF genes as transport-related-genes. Based on MIBiG comparison analysis, NPRS in region 1.4 was closest to genes encoding virginiafactin A, B, C, and D, which are produced by *Pseudomonas* sp. QS1027 (42%). NRPS in region 1.5 is located between 1,500,972 and 1,563,111 nt, which contained condensation and AMP-binding gene as the core biosynthetic gene; PP-binding, SMCOG1127: condensation domain-containing protein, peptidase S41, SMCOG1009: mbtH-like protein, SMCOG1022: beta-ketoacyl synthase, aminotran 1 2, and PF04055 as additional genes; and SMCOG1202: major facilitator transporter as transport-related gene; and SMCOG1136: GntR family transcriptional regulator as regulatory genes. Based on MIBiG comparison analysis, NPRS in region 1.5 was closest in similarity to mutanocyclin produced by *Streptococcus mutans* B04Sm5.

NRPS in region 2.1 consisted of condensation and AMP-binding gene as the core biosynthetic gene; GST C as an additional gene; and SMCOG1003: sensor histidine kinase and SMCOG1112: sigma-54 dependent transcriptional regulator as regulatory genes; and SMCOG1000:ABC transporter ATP-binding protein and SMCOG1029: RND family efflux transporter MFP subunit as transport-related genes. Based on MIBiG comparison analysis, the NRPS in region 2.1 was closest in similarity to taxlllaid A, produced by *Xenorhabdus bovienii* SS-2004. NRPS in region 2.2 consisted of condensation and AMP-binding gene as the core biosynthetic gene; SMCOG1002:AMP-dependent synthetase and ligase, SMCOG1025:diguanylate cyclase, PF07366, SMCOG1091:glutamine-binding lipoprotein glnH, Glycos_transf_2, PF04055, Fer4_12, Glyco_tran_28_C, SMCOG1193:glutathione S-transferase, SMCOG1001:short-chain dehydrogenase/reductase SDR as additional genes; SMCOG1031:LysR family transcriptional regulator and SMCOG1057:TetR family transcriptional regulator as regulatory genes; and SMCOG1082:TonB-dependent siderophore receptor family and SMCOG1031:LysR family transcriptional regulator as transport-related genes. Based on MIBiG comparison analysis, the NRPS in region 2.2 was closest in similarity to the gene that produces 5-fluoro-2,3,4-trihydroxypentanoic acid (24%) produced by *Streptomyces* sp. MA37. NRPS in region 2.7 consisted of 65,461 nt, including the condensation and AMP-binding gene as the core biosynthetic gene; SMCOG1091: glutamine-binding lipoprotein glnH, SMCOG1193: glutathione S-transferase, peptidase_S41, and SMCOG1025: diguanylate cyclase as additional genes; SMCOG1031: LysR family transcriptional regulator, SMCOG1003: sensor histidine kinase, SMCOG1031: LysR family transcriptional regulator as regulatory genes; and SMCOG1202: major facilitator transporter as a transport-related gene. Based on MIBiG comparison analysis, the NRPS in region 2.7 was closest in similarity to the gamexpeptide C gene (48%) in *Photorhabdus raimondii* subsp. raimondii TTO1.

### Class I lanthipeptide (Lant Class I)

The gene for Lant class 1 was encoded on chromosome 1 of *P. viridis* BBR56 (region 1,199,639–1,224,010 nt). Lantibiotics or antibiotics containing lanthionine have antibacterial properties. LANC_like, Lant dehydr N, and Lant_dehydr_C act as the core biosynthetic genes for Lant Class I; SMCOG1053:beta-lactamase, Lanthipeptide_LanB_RRE, SMCOG1155: lantibiotic dehydratase domain protein, NTP_transf_3, SMCOG1064: glucose-1-phosphate adenylyl/thymidylyltransferase, and peptidase_C39 act as additional genes; SMCOG1288:ABC transporter-related protein, SMCOG1029:RND family efflux transporter MFP subunit, and SMCOG1049:AcrB/AcrD/AcrF family protein as transport-related genes. No regulatory gene was detected in this region. MIBiG comparison revealed that Lant class 1 encoded by this genome was closest in similarity to thalassomonasin A and thalassomonasin B (48%) produced by *Thalassomonas actinium* (Table 2).

**Table 2:** MIBiG comparison of *P. viridis* BBR56 for Lant Class I

BGC Reference	Similarity score (%)	Type	Compound	Microorganism
BGC0002640	48	RiPP	Thalassomonasin A and thalassomonasin B	*Thalassomonas actinium*
BGC0002630	37	RiPP	Testisin	*Lysobacter antibioticus*
BGC0001555	36	RiPP	Colicin V	*Escherichia coli chi7122*
BGC0000588	36	RiPP	Microcin L	*Escherichia coli*
BGC0000542	28	RiPP	Penicidin B	*Paenibacillus terrae*
BGC0002005	27	RiPP	RaxX	*Xanthomonas oryzae pv. oryzae*
BGC0000554	27	RiPP	SRO15-3108	*Streptomyces filamentosus* NRRL 15,998
BGC0002698	26	RiPP	Phaeornamide	*P. arcticus* DSM 23,566
BGC0000538	26	RiPP	Nisin Z	*Lactococcus lactis*

### NRP-metallophore, NRPS, T1PKS, betalactone, and thioamide-NRP

**Fig. 7:**
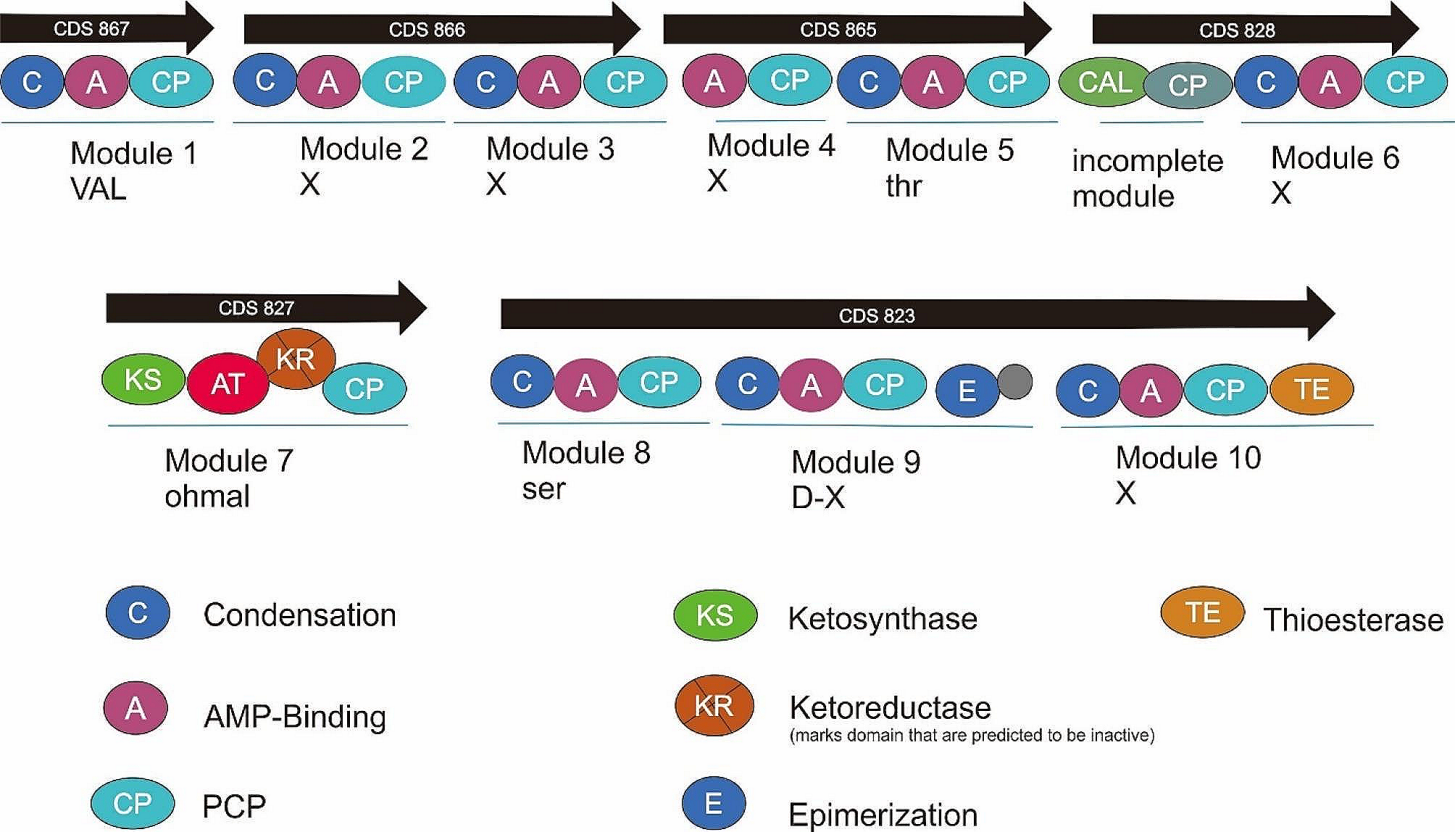
Schematic of the NRP-metallophore, NRPS, T1PKS, betalactone, and thioamide-NRP (CDS 867, 866, 865, 828, 827, and 823) contained in *P. viridis* BBR56

Region 1.2 of this genome contained genes encoding NRP-metallophore, NRPS, T1PKS, betalactone, and thioamide-NRP from 916,452 to 1,043,234 nt (Fig. 7). For NRP-metallophore, EntC was used as the core gene, SMCOG1018: isochorismate synthase was used as an additional gene; and SMCOG1288:ABC transporter-related protein was used as a transport-related gene. For betalactone-thioamide, HMGL-like acted as the core gene; SMCOG1271:2-isopropylmalate synthase as an additional gene; and SMCOG1058: ArsR family transcriptional regulator acted as a regulatory gene for this BGC region. Based on MIBiG comparison of AntiSMASH analysis, genes encoding NRP-metallophore, NRPS, T1PKS, betalactone, and thioamide-NRP was closest in similarity to those encoding taxallid A produced by *Xenorhabdus bovienii* SS-2004, syringafactin A and syringafactin C produced by Pseudomonas sp. *SZ57*, and xenematide produced by *Xenorhabdus nematophila* AN6/1.

### Hydrogen cyanide

Genes involved in the biosynthesis of hydrogen cyanide were detected in chromosome 1 (2,818,632–2,831,565 nt). This BGC region consisted of Fer2_4, Fer2_BFD, Pyr_redox_2, and DAO as the core genes; FAD-dependent oxidoreductase as an additional gene; SMCOG1051: TonB-dependent siderophore receptor as a transport-related gene; and SMCOG1167: transcriptional regulator as a regulatory gene. Based on MIBiG comparison analysis by AntiSMASH, the genes involved in hydrogen cyanide production were closest in similarity to those involved in hydrogen cyanide production by *Pseudomonas fluorescens* (Table 3).

**Table 3:** MIBiG comparison of *P. viridis* BBR56 with hydrogen cyanide and predicted substances

BGC Reference	Similarity score (%)	Type	Compound	Microorganism
BGC0002345	39	Other	Hydrogen cyanide	*Pseudomonas fluorescens*
BGC0002489	14	Other	Pseudopaline	*Pseudomonas aeruginosa* PAO1
BGC0002133	13	RiPP	1 Methanobactin	*Methylosinus* sp. LW3
BGC0000947	8	Other	Vibrioferrin	*Vibrio alginolyticus*
BGC0002087	5	NRP, Polyketide	Gliostatin A	*Burkholderia gladioli* 238

### Prodigiosin

Prodigiosin, a bioactive natural product produced by *Serratia marcescens* and *P. rubra*, was detected in the BBR56 genome. This BGC was located from 4,128,390 to 4,163,412 nt (total 35,023 nt) and consisted of several core, transport-related, and regulatory genes. The core genes of prodigiosin detected in *P. viridis* BBR56 were PPDK_N, PP-binding, AMP-binding, and PKS_KS. Aminotran_1_2, SMCOG1109:8-amino-7-oxononanoate synthase, SMCOG1002: AMP-dependent synthetase and ligase, ketoacyl-synt, SMCOG1022: beta-ketoacyl synthase, SMCOG1006:acyl-CoA dehydrogenase, SMCOG1013:aminotransferase class-III, aminotran 3, SMCOG1042:O-methyltransferase, SMCOG1147:putative acyl carrier protein, SMCOG1012:4ʹ-phosphopantetheinyl transferase, Peptidase_S8, and SMCOG1075:alkaline serine protease; the subtilase family were additional genes. SMCOG1116: homoserine/threonine efflux protein, SMCOG1005: drug resistance transporter, EmrB/QacA, SMCOG1086:MATE efflux family protein were transport-related genes. SMCOG1008: response regulator was a regulatory gene for prodigiosin production. Based on MIBiG analysis by AntiSMASH, the similarity of prodigiosin of *P. viridis* was closest to the di-pyrrolyl-dipyrromethene prodigiosin produced by *P. rubra* (Table 4).

**Table 4:** MIBiG comparison of *P. viridis* BBR56 for prodigiosin and predicted substances

BGC Reference	Similarity score (%)	Type	Compound	Microorganism
BGC0002675	61	Polyketide	Di-pyrrolyl-dipyrromethane Prodigiosin	*Pseudoalteromonas rubra*
BGC0000259	45	Polyketide	Prodigiosin	*Serratia marcescens*
BGC0001137	28	Alkaloid	Marinacarboline A, marinacarboline B, marinacarboline C, marinacarboline D	*Marinactinospora thermotolerans*
BGC0000858	25	Other	Ectoine	*Methylobacter marinus*
BGC0000260	24	Polyketide	Prodigiosin	*Hahella chubuensis* KCTC 2,396

## Discussion

The molecular identification of BBR56 showed that this strain was most similar to *P. viridis* G-1387. The BBR56 isolate produced a red pigment and grew well on media with a salinity of up to 20 ppt. Marine bacteria grow in seawater with a salinity of 20 ppt. The ethyl acetate extract of the cell-free supernatant of BBR56 isolated from seawater inhibited growth of the pathogen *V. harveyi*. In nature, these bacteria can easily be found free or associated with marine organisms and sediments. *V. harveyi* often poses a threat to the mariculture industry, especially shrimp, bivalves, and fish [40]. *V. harveyi* produces a high mortality rate, causing a sharp decline in production. Outbreaks of *V. harveyi* also occur in controlled cultivation environments and are resistant to several types of antibiotics, including oxytetracycline, ampicillin, erythromycin, and kanamycin [17, 41]. Thus, the discovery in this study will support the development of new antibiotics to treat vibriosis in mariculture.

This study revealed many aspects of *P. viridis* that have rarely been studied until now. A thorough investigation of the genes responsible for producing natural compounds is required to facilitate the study and use of this microorganism. WGS analysis is currently the best option for exploring the potency of bacteria using computerized tools at an affordable cost [42]. indicated that WGS can be used to analyze the DNA sequence and base order in the genome of a sample using an automated DNA sequencer and computational method. The WGS analysis in this study used the Oxford Nanopore Technology platform GridION for long read sequencing. This is the fourth-generation technology of DNA sequencing. This technique has several advantages, including being label-free for very long reads, requiring limited samples, and having a high output [43]. We performed BGC studies and analysis from whole genome sequence data using various platforms, including antiSMASH, BAGEL4, RASTk, and Orthovenn. All these platforms are available online and easy to access.

From the WGS data, we conducted genomic comparison analysis for *P. viridis* BBR56 and revealed that the genome size of this bacteria was closest to that of *P. maricaloris* (5.5 Mbp). The smallest of the five genomes compared in this study was *P. piscicida*, 4.2 Mbp, and the largest from *P. rubra*, 6.1 Mbp. Many studies have used WGS to analyze *Pseudoalteromonas* species, namely for *P. tunicata* [12], *Pseudoalteromonas* sp [44]., *P. piscicida* [13], *P. agarivorans* [14], *P. atlantica* [16], *Pseudoalteromonas* sp. CO109Y [45], and *P. xiamenensis* [6], and some of this WGS research has explored and investigated potential secondary metabolite compounds.

Comparison of cluster orthologous genes was determined by Orthovenn2, and several similarities of protein clusters in these five genomes were investigated. Three-quarters of the encoded proteins in the genome were involved in processing metabolism, protein production, energy, and stress response. The components of metabolic products, namely nucleotides, carbohydrates, amino acids, and lipids, are used to produce many important substances and energy during the bacterial life cycle. The byproducts of these mechanisms are normally used for other substrate- producing mechanisms. There are two types of metabolites, primary and secondary metabolites, based on their metabolic pathways and functional properties. According to [46], secondary metabolites are stimulated by stressors in the environment or as a stress response. Secondary metabolites are produced under specific conditions and are not used for functional biological activities such as growth and reproduction. Environmental conditions considerably influence the production of these natural compounds. Marine ecosystems are much more complex than freshwater ecosystems; therefore, the potency of marine resources is more abundant than that of terrestrial resources.

*The Pseudoalteromonas* genus has been extensively researched because these species can produce natural compounds such as the purple pigment violacein and the tryptophan analog indolmycin, which are obtained from *P. luteoviolaceae* S4054 [47]. Prodigiosin genes are present in the *P. rubra* and *P. xiamenensis* genomes [6]. Decatetraenoic acid is produced by *Pseudoalteromonas* sp., which can disrupt *V. alginolyticus* [48]. Unfortunately, information and studies are lacking regarding the secondary metabolites produced by *P. viridis* BBR56. Several online platforms can be used for deeper investigation of various genes in the bacterial genome. Recently, technological developments and advances have advanced genomic analyses, and simplified the exploration of active ingredients. Continued identification of antibiotics or other compounds is urgently needed as an alternative to current antibiotics, which no longer treat pathogenic infections. The BGC analysis of the *P. viridis* BBR56 genome is surprising because this identified genes that produce several potent secondary metabolites, such as NRPS, PKS, RiPP-like, betalactone, hydrogen cyanide, and even prodigiosin.

Ref. [49] reported that PKS and NRPS are often detected in *Pseudoalteromonas* BGCs. The investigation of bioactive compounds and biosynthetic pathways for NRPS and PKS has proven to require advanced techniques [50]. Six regions of NRPS, T3PKS, and T1PKS-like betalactone were identified in this study in the genome of *P. viridis* BBR56. The predicted substance in the antiSMASH analysis that is produced by the NRPS genes is taxlllaid A, which is produced by *Xenorhabdus bovienii* SS-2004. All NRPS genes detected in the genome were similar to those from other bacteria but not *Pseudoalteromonas*. Taxlllaid A-G are natural products produced by *Xenorhabdus* and have activity against *Plasmodium falciparum* [51]. Several NRPS substances have been identified from *Pseudoalteromonas*, namely dibromoalterochromide and bromoalterochromide, which are produced by *P. rubra, P. flavipulchra*, and *P. maricaloris*. A bioactive compound, cyclotetrapeptide, is produced by *P. maricaloris* [4, 52, 53]. All these compounds can inhibit pathogenic bacteria and fungi.

The genome of these bacteria contained the prodigiosin BGC, and this is the first report on its discovery from *P. viridis.* Prodigiosin is a natural product with a red pigmentation and has been successfully isolated from *P. rubra*. This substance has a tripyrrole structure and acts as an antibiotic for several pathogens [54–57] [58]. stated that the first prodiginine was purified from *Serratia marcescens*, and its production has been demonstrated in both marine and freshwater bacteria (*Pseudomonas*, marine *Pseudoalteromonas, Hahella, Vibrio*, and *Zooshikella*). *Pseudoalteromonas* species that contain the prodiginin-prodigiosin genes are *P. rubra, P. deitrificans*, and *P. xiamenensis* [6, 59].

Genes encoding RiPP-like proteins were investigated on chromosomes 1 and 2 of *P. viridis* BBR56. RiPPs belongs to a large family of bioactive substances, including alkaloids, nonribosomal peptides, and terpenoids, which have a high molecular weight, which is estimated at 110 kDa [60]. RiPP-like genes are often found in the genome of *Pseudoalteromonas* species. NRPS and RiPP-like have different enzyme requirements and are multimodular enzyme complexes that incorporate the backbone of a peptide [61]. Gene encoding Lant class I was also detected in the *P. viridis* BBR56 genome. This study could enable the discovery of new lanthipeptides production by using BGCs. Lanthipeptide genes are conserved, and different enzymes other than RiPP are used after modification [62]. Lanthipeptides are ribosomally synthesized cyclic peptides that can be posttranslationally modified [63]. Class I lanthipeptides can disrupt pathogenic bacterial growth and can act as antibiotics. Currently, five classes of lanthipeptides are known. The Lant Class I peptide encoded in the BBR56 genome was most similar to thalassomonasin A–B, which is produced by *Thalassomonas actinium*. Thalassomonasin A can be used as an antifungal agent [64, 65]. *Pseudoalteromonas* is a highly useful genus, especially in the production of secondary metabolites. However, *P. viridis* BBR56 has not been explored on an advanced level. Based on analysis of BGC prediction by genome mining, newly identified bioactive substances, especially antibiotics, may be identified as antibacterial BGCs in the genome. Thus, *P. viridis* BBR56 has potential to produce new marine antibiotics for aquaculture and other purposes.

## Data Availability

The following are information regarding the deposition of whole-genome sequences: The complete genome sequence has been deposited at GenBank under the following accession number Chromosome 1 and Chromosome 2 (CP072425-CP072426), BioProject PRJNA716373, and Biosample SAMN18435505. The raw sequencing reads have been deposited in the Sequence Read Archive (SRA) under accession number SRR14179986.
